# COVID‐19 as an accelerator for digitalization at a German university: Establishing hybrid campuses in times of crisis

**DOI:** 10.1002/hbe2.201

**Published:** 2020-06-19

**Authors:** Alexander Skulmowski, Günter Daniel Rey

**Affiliations:** ^1^ Psychology of Learning with Digital Media Chemnitz University of Technology Chemnitz Germany

**Keywords:** COVID‐19, digitalization, e‐learning, human cognition, human‐computer interaction, hybrid campus, internet, multimodal learning, social media, social networking

## Abstract

As a result of the COVID‐19 outbreak, teaching in universities needed to be quickly transitioned from regular on‐campus classes into technology‐enhanced teaching formats. In this article, we present the case study of Chemnitz University of Technology (Germany), where digital classes were introduced in a matter of weeks. By analyzing syllabus data, we found that the use of video and video conferencing is an important current development. Related to these findings, we present evidence from instructional psychology and social media research that can help in the design of teaching during this crisis. We highlight the need for multimodal learning, that is, learning settings that use multiple sensory modalities. Importantly, we present a strategy of *hybrid campuses* for this and potential future emergencies. This approach describes how the social distancing measures currently in effect can be used to re‐think higher education based on a reasonable use of technology. Taken together, the COVID‐19 crisis can be a time of major reform in higher education that will accelerate the process of digitalization in an unprecedented way.

## 1 INTRODUCTION

The outbreak of COVID‐19 in Germany required urgent changes to the plans for the summer term at German universities that have the potential to fundamentally transform teaching for the foreseeable future. Although technology‐enhanced learning already played an important part in most German universities, the COVID‐19 pandemic has required to expand the existing infrastructure and highlighted areas that universities should focus on in their digitalization strategy. Furthermore, this challenge has greatly accelerated the digitalization of the German higher education sector. However, it needs to be highlighted that most schools and some universities in Germany are not adequately equipped to handle the current transformation towards fully‐digital forms of education (Kerres, 2020). Furthermore, most forms of technology‐enhanced learning in German universities are limited to auxiliary services, such as providing lecture slides or literature as a digital supplement to real‐life classes. Importantly, this crisis required substantial efforts in creating new infrastructure to enable more demanding forms of technology‐enhanced learning such as video conferencing (Kerres, 2020). In this article, we present how one German university, Chemnitz University of Technology, has handled the transition from on‐campus teaching to technology‐enhanced learning at the onset of the COVID‐19 crisis. By analyzing the syllabus of the digital classes, we highlight developments in the design and choice of teaching formats. Taken together with a discussion of recent studies on the effects of the changes in social interactions and well‐being associated with internet use, we devise guidelines for assuring successful higher education in (potentially re‐occurring) lockdown situations.

## 2 CASE STUDY

### 2.1 Case context

With its 10,389 students (winter term 2019–2020; Chemnitz University of Technology, 2020b), Chemnitz University of Technology can be considered a middle‐sized university and, therefore, shares several current challenges with most other European and international universities. Chemnitz University has a focus on STEM education with faculties in Computer Science, Mechanical Engineering, Natural Sciences, and other fields, but also features faculties in the Humanities and Behavioral and Social Sciences. Due to this range of bachelor's and master's courses, the conclusions from this case study should be informative for most universities. Regular on‐campus classes were originally set to begin on April 6, 2020 (Chemnitz University of Technology, 2020a).

### 2.2 Measures taken by the university

In response to several measures taken by the German government against the COVID‐19 pandemic, the Rector of the university regularly published Open Letters in which details concerning the operations of the university were given (Strohmeier, 2020). The third Open Letter from March 17, 2020, contained an announcement that teaching formats were being considered that do not require the physical attendance of students. On March 20, an Open Letter contained an announcement that a list of digital classes would be released in April. On April 1, a list of over 700 digital classes was provided and additional classes were entered into this list in the following days. This summary of the events demonstrates the short time in which classes were transitioned from on‐campus to digital.

Additional measures taken by the university include the establishment of a psychological counseling hotline and several technical changes. One of the most important new tools introduced during this time is the setup of the video conference software BigBlueButton (https://bigbluebutton.org).

When browsing through the syllabus (Chemnitz University of Technology, 2020c), it becomes apparent that nearly half of all course descriptions have been edited to include information regarding whether and how the course will use a digital format or technology‐based enhancements. In the following, we will analyze the syllabus data more closely to gain an understanding of current developments in the use of technology‐enhanced learning.

### 2.3 Syllabus data

We analyzed the syllabus of Chemnitz University of Technology for the summer term 2020 (Chemnitz University of Technology, 2020c) as retrieved on April 16, 2020, for each of the eight faculties and the Center for Teacher Training (ZLB). The university asked the teaching staff to answer a form regarding whether each class will be held as a digital class and permitted to add a note to each entry in the syllabus. We analyzed these notes to find out whether there are preferences in the use of technology‐enhanced teaching formats depending on faculty (see Table 1). Out of the 2,256 classes offered at Chemnitz University of Technology, 1,051 were confirmed to take place digitally partly or in full. Furthermore, we analyzed the number of digital classes that explicitly mentioned the use of a number of technology‐based teaching tools (BigBlueButton, video, forum, and chat) in their syllabus description. To be counted, descriptions needed to contain the keywords “BigBlueButton” (or different synonyms and abbreviations such as “BBB”), “video” (excluding classes that only used BigBlueButton), “forum,” or “chat” (excluding video chats).

**TABLE 1:** Descriptive statistics of classes and keywords found in the syllabus

Faculty	Total classes	Digital classes	BigBlueButton	Video (excl. BigBlueButton)	Forum	Chat (excl. video)
Natural Sciences	277	56	0	10	0	0
Mathematics	157	49	0	15	3	2
Mechanical Engineering	379	192	4	13	6	0
Electrical Engineering and Information Technology	235	107	18	40	13	0
Computer Science	176	98	3	22	3	0
Economics and Business Administration	381	130	0	15	0	0
Humanities	282	206	3	10	6	3
Behavioral and Social Sciences	263	131	1	14	9	2
ZLB	106	82	1	7	0	0

An important result is that conventional, text‐based forms of communication and teaching were rarely mentioned in the syllabus notes. The design of only 40 classes appears to make significant use of forums, while only seven classes were described to rely on (non‐video) chats. On the other hand, 146 classes were stated to make use of video‐based teaching formats (including recorded lectures, streams, video conferences, video chats, and video podcasts). Besides, 30 classes aim at using the newly introduced BigBlueButton video conference service of Chemnitz University of Technology. Interestingly, the video‐based formats are particularly often used within the Faculty of Electrical Engineering and Information Technology. Although the descriptions provided in the syllabus are not necessarily exhaustive and may have omitted more traditional tools such as forums and chat‐rooms despite being used in digital classes, these numbers give us an impression for the heightened demand of video‐based forms of teaching.

## 3 DISCUSSION

As described in this case study, the COVID‐19 crisis required a swift response in the transition from on‐campus classes to digital teaching formats. In a matter of days, teaching concepts needed to be made compatible with the emergency situation. Our case study revealed that there is a high interest in the usage of video technology. Several classes feature video recordings, streams, and video conferences. The newly introduced open‐source video conference system BigBlueButton already elicits a high demand.

These results show that the COVID‐19 crisis has already proven to be a significant accelerator for the digitalization of teaching at this particular university. The dire situation has been understood as an opportunity for change and innovation (Yan, 2020). When considering the fact that only a few years ago, many German universities required mandatory attendance of university classes, this drastic increase in flexibility is even more remarkable. As we can only evaluate the success of the measures that were taken at the end of the term, we wish to present a few guidelines for the on‐going crisis based on existing research on digital learning and social media in the following.

### 3.1 Multimodal learning in hybrid campuses

The transformation of university courses from regular on‐campus classes to e‐learning courses in a very short time frame has been a challenge due to the uncertainty regarding the starting date for regular on‐campus lectures and classes. As a result of the lockdown policy of the German government and the federal state of Saxony, it was unclear if and when regular teaching could commence even at the start of the teaching period in April. Thus, most classes have been modified to start with a digital phase that may span the entire term or that can be supplemented with on‐campus classes, meetings, or other forms of face‐to‐face contact. We refer to this new teaching model as *hybrid campuses*. This emerging strategy of combining digital and face‐to‐face teaching appears to be a promising choice for the following months and years in which additional waves of COVID‐19 and related infections may spread rapidly and unexpectedly all over the globe. Before the COVID‐19 crisis, the teaching format of *blended learning* was already in use (for an analysis, see Moskal, Dziuban, & Hartman, 2013). However, the concept of blended learning, which can have several meanings that usually imply a combination of real‐life and digital teaching components, has been criticized as being too vague (Oliver & Trigwell, 2005). As we will discuss at a later stage, the social distancing measures of the COVID‐19 crisis require a more precise model that aims at maximizing interpersonal contact as permitted by social distancing regulations while strategically using new and emerging technology.

As can be seen from the measures that were taken, an emerging field for the increased use of digital technologies is *multimodal learning*, that is, learning settings that target more than one sensory modality (for an overview, see Skulmowski & Rey, 2018). While previous forms of online learning (and blended learning) focused on providing literature, supplying tasks and tests as well as offering support (usually through text messages), multimodal forms of learning and teaching are getting increasingly relevant. A recent paper on the effects of COVID‐19 on a Chinese university emphasized often neglected factors such as speakers' voices, their body language, and other social aspects that can hardly be transmitted via common text‐based forms of e‐learning (Bao, 2020). In order to find a viable long‐term solution, we argue that technology‐enhanced learning requires a holistic approach that incorporates bodily, physical, and social aspects of learning. Simply put, we argue that the social life found in a university cannot adequately be reduced to a series of direct message exchanges. Yet, tools that offer a digitalization of the multimodal and social nature of learning are only just emerging. The high interest in video technology can be taken as an indicator for the interest in multimodal learning. In the following section, we will discuss potential extensions beyond videos and streaming.

### 3.2 Optimizing learning through emerging technologies

When looking more closely at the classes in the syllabus of Chemnitz University of Technology that were not switched to a digital format, we noticed that some of these were more practical classes that require specialized equipment and laboratories. We are convinced that the next step in the digitalization of higher education should include these kinds of courses. For instance, there is a large body of research in the field of medical education investigating emerging technologies such as virtual reality (Jensen & Konradsen, 2018), augmented reality (Barsom, Graafland, & Schijven, 2016), and mixed reality (Skulmowski, Pradel, Kühnert, Brunnett, & Rey, 2016). It is also possible to substitute costly computer equipment with smartphone‐based solutions (Lee, Sergueeva, Catangui, & Kandaurova, 2017). With the increasing functionality of the Internet of Things (for an overview, see Paul & Jeyaraj, 2019), several other sources of sensor‐based information could be harnessed. Importantly, this historic turn could also be the time in which artificial intelligence is more strongly integrated into technology‐enhanced learning, for instance as a system that detects learning progress and adapts the difficulty of contents to the learner's skill level (Songer, Newstadt, Lucchesi, & Ram, 2019).

However, we want to emphasize that not all courses need to be transformed into a multimodal digital format. For example, not all introductory classes in which basic concepts are taught need immersive virtual reality environments. The aim should be to provide students with a learning setting that optimally prepares them for their tests and their later vocation. If the latter heavily rely on manual tasks, the use of specific physical machinery, or social competencies, universities should utilize sufficient technological means in their hybrid campuses to train such abilities. The existing literature on instructional psychology and education offers a wealth of research that can be used as starting points for cost–benefit analyses.

More sophisticated forms of hybrid campuses may be based on a partial digitalization that prominently makes use of technology in the learning process as outlined above, but still contains several opportunities for face‐to‐face interaction. During the on‐going COVID‐19 crisis, these real‐life social contacts might consist of meet‐ups of small groups of students that are digitally connected with their peers. Depending on the severity of the social distancing measures, these encounters can be extended or limited as needed.

### 3.3 Avoiding pitfalls

Importantly, these new ways of connecting people through digital technology should cautiously acknowledge some of the negative effects that are considered to be the results of social media use. A recent experimental study found that a reduced usage of a popular social network increased well‐being, fostered a healthy lifestyle, and reduced symptoms of depression (Brailovskaia, Ströse, Schillack, & Margraf, 2020). A comparison of teachers' and parents' assessment of children's social skills found that there has not been a major decline in these skills between 1998 and 2010 (Downey & Gibbs, 2020). However, the social skills of children that spent more time on social networks and in online games were rated as lower (Downey & Gibbs, 2020). It is important to consider these potential effects on social life given the decrease of face‐to‐face social contacts in younger populations. Twenge and Spitzberg (2020) describe that for Americans between the age of 15 and 25, the duration of “non‐digital” social interactions decreased by 21 to 23 minutes between 2003 and 2017. Taken together, the existing evidence suggests that even small changes in the daily life of young people can have a substantial effect. Therefore, an important lesson to learn from the presented evidence is that a narrow focus on introducing more “social” features in e‐learning systems may be a facile approach. We do not wish to imply that digital social networking features necessarily affect young people in a negative manner, but it becomes clear that the effects of large‐scale changes in the daily life of entire populations need to be closely monitored. Furthermore, the presented studies can be taken as indications that social media alone may not be a sufficient substitute for real‐life social contacts. Therefore, we argue for a stronger emphasis on integrating real‐life meetings while offering the option to communicate with larger groups via technology‐based means.

Furthermore, there have been several findings suggesting that the use of some forms of multimedia learning is not always beneficial for learning. Technologies such as virtual reality can in some cases be an obstacle in the learning process because the feeling of immersion (i.e., the impression of existing in a virtual world) can actually induce cognitive load by itself (Makransky, Terkildsen, & Mayer, 2019). Similarly, interactive learning media allow a responsive design of instruction, yet interactivity has also been found to be a cause of cognitive load (e.g., Skulmowski & Rey, 2020). Hence, these technologies should only be used if there is a clear purpose that cannot be achieved otherwise and not solely to make learning more entertaining.

Besides, various other findings run counter to educators' intuitions. As we have seen in our case study, videos and video conferencing appear to be important new components of digital classes. However, a large‐scale investigation found that seeing the instructor's face in an instructional video does not affect learning (Kizilcec, Bailenson, & Gomez, 2015). This is an example of the fallacy of attempting to faithfully reproduce real‐world classes in a digital format and shows that the digitalization process requires university educators to re‐think their digital teaching. The goal should not be to re‐create the “ritual” of classes in a digital form but to find efficient ways of providing students with the information they need and opportunities to apply this knowledge.

Budgetary limits and legal hurdles can severely impact technology‐enhanced learning (see Kerres, 2020). For instance, data protection laws and a lack of funding can affect the choice of video conferencing software (Kerres, 2020). As a result, the use of open source software such as BigBlueButton that can be self‐hosted by universities without the need for external providers may be a viable alternative.

## 4 CONCLUSION

While the sudden and unexpected outbreak of COVID‐19 required university lecturers and administrators to quickly adapt to a new situation, the data presented in this case study provide evidence that most university courses can be rapidly transformed into a digital format. Given the fact that there may be some heterogeneity concerning the experience with technology‐enhanced learning throughout the teaching staff, it will be interesting to assess how well less experienced users of e‐learning services have dealt with this emergency situation. In any event, this situation has greatly accelerated the digitalization of university teaching and may contribute to the permanent establishment of hybrid campuses that offer flexibility and autonomy for students as well as educators. Time will tell whether university educators who switched to online teaching formats merely consider technology‐enhanced learning as the second‐best alternative to face‐to‐face on‐campus classes or whether they use this situation to re‐invent teaching.

## CONFLICT OF INTEREST

The authors declare no potential conflict of interest.
